# Entropy-Based Clustering Algorithm for Fingerprint Singular Point Detection

**DOI:** 10.3390/e21080786

**Published:** 2019-08-12

**Authors:** Ngoc Tuyen Le, Duc Huy Le, Jing-Wein Wang, Chih-Chiang Wang

**Affiliations:** 1 Institute of Photonic Engineering, National Kaohsiung University of Science and Technology, Kaohsiung 80778, Taiwan; 2 Department of Electronic Engineering, National Kaohsiung University of Science and Technology, Kaohsiung 80778, Taiwan; 3 Department of Computer Science and Information Engineering, National Kaohsiung University of Science and Technology, Kaohsiung 80778, Taiwan

**Keywords:** singular point detection, boundary segmentation, blurring detection, fingerprint image enhancement, fingerprint quality

## Abstract

Fingerprints have long been used in automated fingerprint identification or verification systems. Singular points (SPs), namely the core and delta point, are the basic features widely used for fingerprint registration, orientation field estimation, and fingerprint classification. In this study, we propose an adaptive method to detect SPs in a fingerprint image. The algorithm consists of three stages. First, an innovative enhancement method based on singular value decomposition is applied to remove the background of the fingerprint image. Second, a blurring detection and boundary segmentation algorithm based on the innovative image enhancement is proposed to detect the region of impression. Finally, an adaptive method based on wavelet extrema and the Henry system for core point detection is proposed. Experiments conducted using the FVC2002 DB1 and DB2 databases prove that our method can detect SPs reliably.

## 1. Introduction

**Figure 1:**
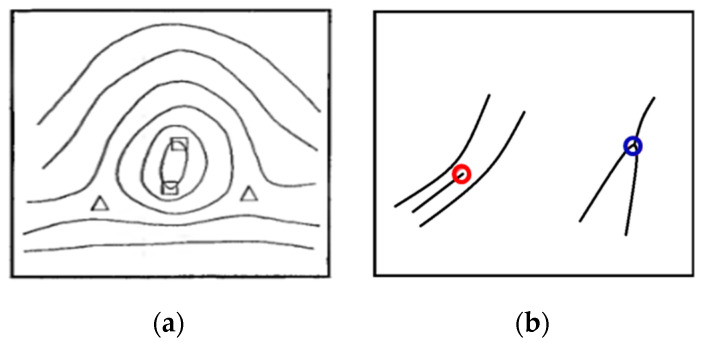
The global and local features in the fingerprint. (**a**) Singular points (SPs) (square: core; triangle: delta) and (**b**)minutiae (red circle: ridges ending; blue circle: bifurcation).

Fingerprint biometrics is increasingly being used in the commercial, civilian, physiological, and financial domains based on two important characteristics of fingerprints: (1) fingerprints do not change with time and (2) every individual’s fingerprints are unique [1,2,3,4,5]. Owing to these characteristics, fingerprints have long been used in automated fingerprint identification or verification systems. These systems rely on accurate recognition of fingerprint features. At the global level, fingerprints have ridge flows assembled in a specific formation, resulting in different ridge topology patterns such as core and delta (singular points (SPs)), as shown in Figure 1a. These SPs are the basic features required for fingerprint classification and indexing. Local fingerprint features are carried by local ridge details such as ridge endings and bifurcations (minutiae), as shown in Figure 1b. Fingerprint minutiae are often used to conduct matching tasks because they are generally stable and highly distinctive [6].

Most previous SP extraction algorithms were performed directly over fingerprint orientation images. The most popular method is based on the Poincaré index [7], which typically computes the accumulated rotation of the vector field along a closed curve surrounding a local point. Wang et al. [8] proposed a fingerprint orientation model based on 2D Fourier expansions to extract SPs independently. Nilsson and Bigun [9] as well as Liu [10] used the symmetry properties of SPs to extract them by first applying a complex filter to the orientation field in multiple resolution scales by detecting the parabolic and triangular symmetry associated with core and delta points. Zhou et al. [11] proposed a feature of differences of the orientation values along a circle (DORIC) in addition to the Poincaré index to effectively remove spurious detections, take the topological relations of SPs as a global constraint for fingerprints, and use the global orientation field for SP detection. Chen et al. [12] obtained candidate SPs by the multiscale analysis of orientation entropy and then applied some post-processing steps to filter the spurious core and delta points.

However, SP detection is sensitive to noise, and extracting SPs reliably is a very challenging problem. When input fingerprint images have poor quality, the performance of these methods degrades rapidly. Noise in fingerprint images makes SP extraction unreliable and may result in a missed or wrong detection. Therefore, fingerprint image enhancement is a key step before extracting SPs.

Fingerprint image enhancement remains an active area of research. Researchers have attempted to reduce noise and improve the contrast between ridges and valleys in fingerprint images. Most fingerprint image enhancement algorithms are based on the estimation of an orientation field [13,14,15]. Some methods use variations of Gabor filters to enhance fingerprint images [16,17]. These methods are based on the estimation of a single orientation and a single frequency; they can remove undesired noise and preserve and improve the clarity of ridge and valley structures in images. However, they are not suitable for enhancing ridges in regions with high curvature. Wang and Wang [18] first detected the SP area and then improved it by applying a bandpass filter in the Fourier domain. However, detecting the SP region when the fingerprint image has extremely poor quality is highly difficult. Yang et al. [19] first enhanced fingerprint images in the spatial domain with a spatial ridge-compensation filter by learning from the images and then used a frequency bandpass filter that is separable in the radial- and angular-frequency domains. Yun and Cho [20] analyzed fingerprint images, divided them into oily, neutral, and dry according to their properties, and then applied a specific enhancement strategy for each type. To enhance fingerprint images, Fronthaler et al. [21] used a Laplacian-like image pyramid to decompose the original fingerprint into subbands corresponding to different spatial scales and then performed contextual smoothing on these pyramid levels, where the corresponding filtering directions stem from the frequency-adapted structure tensor. Bennet and Perumal [22] transformed fingerprint images into the wavelet domain and then used singular value decomposition (SVD) to decompose the low subband coefficient matrix. Fingerprint images were enhanced by multiplying the singular value matrix of the low-low(LL) subband with the ratio of the largest singular value of the generated normalized matrix with mean of 0 and variance of 1 and the largest singular value of the LL subband. However, the resulting images were sometimes uneven. This is because SVD was applied only to the low subband and a generated normalized matrix was used. To overcome this problem, Wang et al. [23] introduced a novel lighting compensation scheme involving the use of adaptive SVD on wavelet coefficients. First, they decomposed the input fingerprint image into four subbands by 2D discrete wavelet transform (DWT). Subsequently, they compensated fingerprint images by adaptively obtaining the compensation coefficients for each subband based on the referred Gaussian template.

The aforementioned methods for enhancing fingerprint images can reduce noise and improve the contrast between ridges and valleys in the images. However, they are not really effective with fingerprint images having very poor quality, particularly blurring. To overcome this problem, we need to segment the fingerprint foreground with the interleaved ridge and valley structure from the complex background with non-fingerprint patterns for more accurate and efficient feature extraction and identification. Many studies have investigated segmentation on rolled and plain fingerprint images. Mehtreet al. [24] partitioned a fingerprint image into blocks and then performed block classification based on gradient and variance information to segment fingerprint images into blocks. This method was further extended to a composite method [25] that takes advantage of both the directional and the variance approaches. Zhang et al. [26] proposed an adaptive total variation decomposition model by incorporating the orientation field and local orientation coherence for latent fingerprint segmentation. Based on a ridge quality measure that was defined as the structural similarity between the fingerprint patch and its dictionary-based reconstruction, Cao et al. [27] proposed a learning-based method for latent fingerprint image segmentation.

This study proposes an efficient approach by combining the novel adaptive image enhancement, compact boundary segmentation, and a novel clustering algorithm by integrating wavelet frame entropy with region growing to evaluate the fingerprint image quality so as to validate the SPs. Experiments were conducted on FVC2002 DB1 and FVC2002 DB2 databases [28]. The experimental results indicate the excellent performance of the proposed method.

The rest of this paper is organized as follows. Section 2 introduces the proposed image enhancement, precise boundary segmentation, and blurring detection based on wavelet entropy clustering algorithm. Section 3 describes the proposed algorithm for SP detection. Section 4 presents experimental results to verify the proposed approach. Finally, Section 5 presents the conclusions of this study.

## 2. Blurring Detection for Fingerprint Impression

### 2.1. Fingerprint Background Removal

SVD has been widely used in digital image processing [29,30,31]. Without loss of generality, we suppose that *f* is a fingerprint image with a resolution of *M × N* (*M ≥ N*). The SVD of a fingerprint image *f* can be written as follows:

(1) $$f=U\Sigma V^{T},$$

where $U=\left[u_{1},u_{2},\ldots ,u_{N}\right]$ and $V=\left[v_{1},v_{2},\ldots ,v_{N}\right]$ are orthogonal matrices containing singular vectors and Σ=[D,O] contains the sorted singular values on its main diagonal. $D=\mathrm{diag}\;\left(\lambda_{1},\lambda_{2},\ldots ,\lambda_{k}\right)$ with singular values $\lambda_{i},\;i=1,2,\ldots ,k$ in a non-increasing order, O is a M×(M−k) zero matrix, and *k* is the rank of f. We also can expand the fingerprint image as follows:

(2) $$f=\lambda_{1}u_{1}v_{1}^{T}+\lambda_{2}u_{2}v_{2}^{T}+\ldots +\lambda_{k}u_{k}v_{k}^{T},\;\;\lambda_{1}\geq \lambda_{2}\geq \ldots \geq \lambda_{k}.$$

The terms $\lambda_{i}u_{i}v_{i}^{T}$ containing the vector outer-product in Equation (2) are the principal images. The Frobenius norm of the fingerprint image is preserved in SVD transformation:

(3) $${\left\|f\right\|}_{F}^{2}=\overset{k}{\underset{i=1}{\sum }}\lambda_{i}^{2}.$$

Equation (3) shows how the signal energy of f can be partitioned by the singular values in the sense of the Frobenius norm. It is common to discard the small singular values in SVD to obtain matrix approximations whose rank equals the number of remaining singular values. Good matrix approximations can always be obtained with a small fraction of the singular values. The highly concentrated property of SVD helps remove background noise from the foreground ridges.

**Figure 2:**
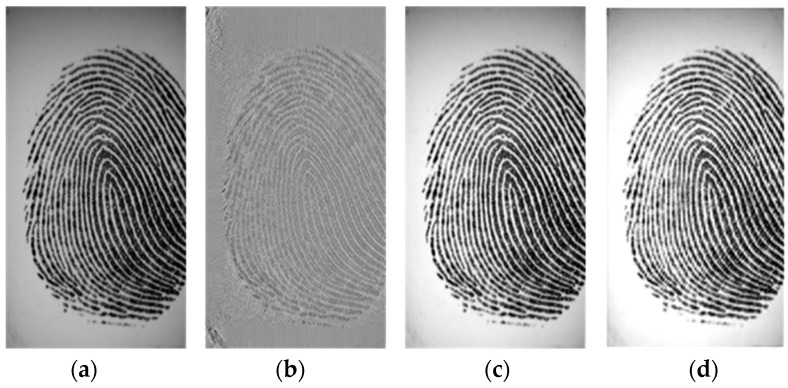
Effects of singular values on a fingerprint image. (**a**) Fingerprint image in FVC 2002 DB2 database; (**b**) reconstructed fingerprint image when all singular values of Figure 2a are set to 1; (**c**) reconstructed fingerprint image when all singular values of Figure 2a are multiplied by 2; (**d**) equalized fingerprint images of Figure 2a.

We performed some experiments to observe the effects of singular values on a fingerprint image. Figure 2a shows a fingerprint image in the FVC 2002 DB2 database. First, all singular values of the fingerprint image, as shown in Figure 2a, were set to 1 and the fingerprint image was then reconstructed. Figure 2b shows the reconstructed fingerprint image without the effect of singular values, implying that the singular vectors represent the background information of the given fingerprint image. Next, all singular values of the fingerprint image shown in Figure 2a were multiplied by 2 and the fingerprint image was then reconstructed. As shown in Figure 2c, the fingerprint image looks clearer and the background of the fingerprint image was removed. It suggests that the singular values represent the foreground ridges of the given fingerprint image. Thus, SVD can be used for enhancing the ridge structure and removing noise from the background of the fingerprint image. In addition, if the fingerprint image is a low-contrast image, this problem can be corrected by replacing Σ with an equalized singular matrix obtained from a normalized image, which is considered to be that with a probability density function involving a Gaussian distribution with a mean and variance calculated using the available dataset. This normalized image is called a Gaussian template image.

Based on observations of the effects of SVD on a fingerprint image, and to effectively remove the background, we examined the singular values of the fingerprint image, which contains most of the foreground information. We automatically adjusted the illumination of an image to obtain an equalized image that has a normal distribution. If the fingerprint image had low contrast, the singular values were multiplied with a scalar larger than 1. A normalized intensity image with no illumination problem can be considered an image that has a Gaussian distribution and that can easily be obtained by generating random pixel values with Gaussian distribution. Moreover, the first singular value contributes 99.72% of energy to the original image and the first two singular values contribute 99.88% of the total energy [31]. The larger singular value represents the energy of the fingerprint pattern and the smaller one, the energy of the background and noise. To effectively remove the background, we set a compensation weight, α, that enhanced the image contrast. It is easy to remove the ridge of images when the compensation weight is larger than 1, and the image contrast is reduced when the compensation weight is smaller than 1. Therefore, we compared the maximum singular value of the Gaussian template with the maximum singular value of the original fingerprint image to compute the compensation weight as follows:

(4) $$\alpha =\{\begin{array}{cc} max\left(\frac{\max\left(\Sigma_{G}\right)}{\max\left(\Sigma \right)},\frac{\max\left(\Sigma \right)}{\max\left(\Sigma_{G}\right)}\right) & ,\;\;\max\left(\Sigma \right)<\eta \\ 1 & ,\;\;max\left(\Sigma \right)\geq \eta \end{array},$$

where the threshold value η is experimentally set as 90,000, and $\Sigma_{G}$ is the singular value matrix of the Gaussian template image with mean and variance calculated from the adopted database as shown in Table 1. The equalized image, $f_{eq}$, having the same size as the original fingerprint image can be generated by the following:

(5) $$f_{eq}=U\left(\alpha \Sigma \right)V^{T}.$$

This task that actually equalizes the fingerprint image can eliminate the undesired background noise. As shown in Figure 2d, the background of the fingerprint image has been removed, thereby providing an image with nearly normal distribution. It also improves the clarity and continuity of ridge structures in the fingerprint image.

### 2.2. Impression Region Detection and Boundary Segmentation

**Figure 3:**
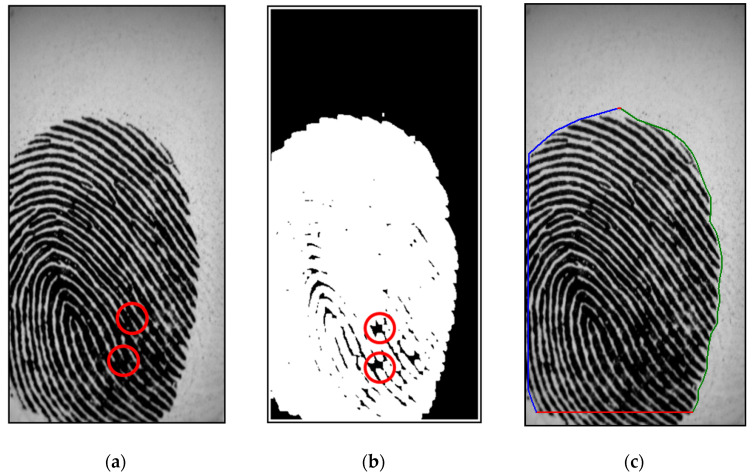
(**a**) Original fingerprint image in FVC 2002 DB2 database; (**b**) binary image by using energy transformation and blur detection obtained with2D non-separable wavelet entropy filtering for Figure 3a; (**c**) segmented image of Figure 3a.

The fingerprint texture should be distinguished from the background by a suitable binary threshold obtained from the energy analysis as a very useful and distinctive preprocessing for boundary segmentation. An analysis of the energy distribution of fingerprint images from the public fingerprint image database indicates a prominent distinction between the fingerprint object and the undesired background owing to the construction of ridges and valleys. In this section, we propose an impression region detection approach based on the energy difference between the impression contour and the background scene. The most obvious feature of the fingerprint ridge is the texture; it exhibits variances in the energy roughness of the impression region. Roughness corresponds to the perception that our sense of touch can feel with an object, and it can be characterized in two-dimensional scans by depth (energy strength) and width (separation between ridges). Before ridge object extraction, a smoothing filter is used to smooth the image and enhance the desired local ridge. The local standard average *µ* and energy *ε* of the 7 × 7 pixels defined by the mask are given by the following expressions:

(6) $$\mu \left(x,\;y\right)=\frac{1}{N}\overset{3}{\underset{i=-3}{\sum }}\overset{3}{\underset{j=-3}{\sum }}f_{eq}\left(x+i,\;y+j\right),$$

(7) $$\varepsilon \left(x,\;y\right)=\frac{1}{N}\overset{3}{\underset{i=-3}{\sum }}\overset{3}{\underset{j=-3}{\sum }}{\left(f_{eq}\left(x+i,\;y+j\right)-\mu \left(x,\;y\right)\right)}^{2},$$

where $f_{eq}\left(x,\;y\right)$ is the equalized image, as discussed in Section 2.1, and *N* = 49 is a normalizing constant. For transforming the grayscale intensity image in Figure 3a into a logical map, a binarized image of the equalized image, $f_{b}\left(x,\;y\right)$, is obtained by extracting the interesting object from the background as follows:

(8) $$f_{b}\left(x,y\right)=\{\begin{array}{cc} 255, & if\;\varepsilon \left(x,\;y\right)\geq 255\\ 0, & if\;\varepsilon \left(x,\;y\right)<255 \end{array},$$

where $f_{b}\left(x,\;y\right)$ is a binarized image; pixel values labeled 255 are objects of interest, whereas pixel values labeled 0 are undesired ones.

Figure 3b shows the binarized image obtained by applying Equation (8) to the equalized image. Based on the binary images, as shown in Figure 3b, we can detect the region of impression (ROI), which is very useful as a distinctive preprocessing of boundary segmentation. Figure 3b shows that the proposed algorithm can perform very well for discriminating the blur region. Pixel (*x, y*) with energy *ε*(*x,y*) ≥ 255 is an object of the ROI; therefore, we can detect the ROI, $f_{ROI}\left(x,\;y\right)$, as follows:

(9) $$f_{ROI}=\left\{\left(x,y\right)|\varepsilon \left(x,y\right)\geq 255\right\}.$$

To define the fingerprint contour, we determine the boundary location of the fingerprint. Most human fingerprint contours have elliptical shapes. Thus, the left, right, and horizontal projections for an elliptical fingerprint contour are divided to search for landmarks by commencing from two sides in every 15 pixels from top to down. Based on the located landmarks, the contour of the fingerprint is acquired in a polygon. As illustrated in Figure 3c, the blue, green, and red lines present the contours received by using left, right, and horizontal projections, respectively. This method is advantageous because it is simple and is less influenced by finger pressure.

### 2.3. Blurring Detection

Our proposed method improves the fingerprint image quality, as discussed in Section 2.1, and the ROI is defined, as discussed in Section 2.2. However, the fingerprint image still contains a blur region within the ROI, leading to the false detection of SPs. In this section, we propose a method for detecting the blur region in a fingerprint image and then ignoring it during detection to reduce the time and improve the accuracy of SP detection.

**Figure 4:**
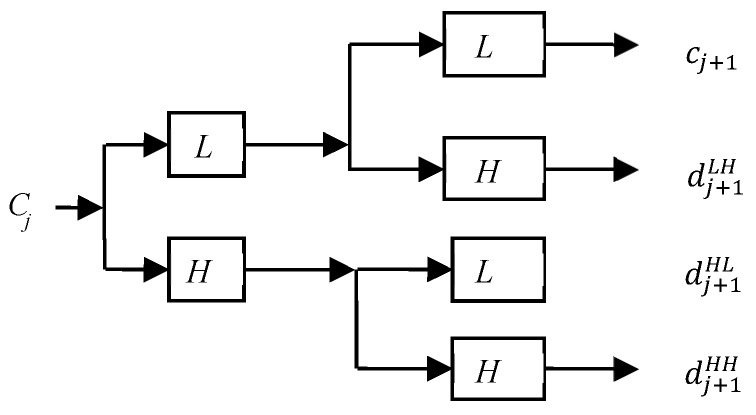
Filter bank implementation of 2D non-separable discrete wavelet transform (NSDWT), *j*: level.

To locate the blur region, we perform region segmentation by finding a meaningful boundary based on a point aggregation procedure. Choosing the center pixel of the region is a natural starting point. Grouping points to form the region of interest, while focusing on 4-connectivity, would yield a clustering result when there are no more pixels for inclusion in the region. After region growing, the region is measured to determine the size of the blur region. Entropy filtering for blur detection of pixels in the 11 × 11 (*N* = 11) neighborhood defined by the mask is given by the following:

(10) $$e_{NSDWF}=\frac{-1}{N^{2}}\overset{N-1}{\underset{x,\;y=0}{\sum }}\left|d^{HH}\left(x,\;y\right)\left|log\right|d^{HH}\left(x,\;y\right)\right|,$$

where $d^{HH}$ is the coefficient of a non-subsampled version of the 2D non-separable discrete wavelet transform (NSDWT) [32,33] in the high-frequency subband decomposed at the first level ($d_{j+1}^{HH}$), *j* = 0, as shown in Figure 4. Figure 3b shows that the proposed algorithm can perform very well for discriminating the blur region.

## 3. SP Detection

In general, SPs of a fingerprint are detected by a Poincaré index-based algorithm. However, the Poincaré index method usually results in considerable spurious detection, particularly for low-quality fingerprint images. This is because the conventional Poincaré index along the boundary of a given region equals the sum of the Poincaré indices of the core points within this region, and it contains no information about the characteristics and cannot describe the core point completely. To overcome the shortcoming of the Poincaré index method, we propose an adaptive method based on wavelet extrema for core point detection. Wavelet extrema contain information on both the transform modulus maxima and minima in the image, considered to be among the most meaningful features for signal characterization.

First, we align the ROI based on the Poincaré’s core points and the local orientation field. The Poincaré index at pixel (*x*,*y*), which is enclosed by 12 direction fields taken in a counterclockwise direction, is calculated as follows:

(11) $$Poincare\left(x,y\right)=\frac{1}{2\pi }\overset{M-1}{\underset{k=0}{\sum }}\Delta \left(k\right),$$

where

(12) $$\Delta \left(k\right)=\{\begin{array}{c} \delta \left(k\right),\;\;if\;\left|\delta \left(k\right)\right|<\pi /2\\ \pi +\delta \left(k\right),\;\;if\;\left|\delta \left(k\right)\right|<-\pi /2\\ \pi -\delta \left(k\right),\;\;\;\;\;\;\;\;otherwise \end{array}$$

and

(13) $$\delta \left(k\right)=\theta \left(x\left(k^{\prime }\right),y\left(k^{\prime }\right)\right)-\theta \left(x\left(k\right),y\left(k\right)\right);\;k^{\prime }=\left(k+1\right)\;\mathrm{mod}\;M;\;M=12,$$

where $\left(x\left(k^{\prime }\right),y\left(k^{\prime }\right)\right)$ and (x(k),y(k)) are the paired neighboring coordinates of the direction fields. A core point has a Poincaré index of +1/2. By contrast, a delta point has a Poincaré index of -1/2. The core pointsdetected in this step are called rough core points.

**Figure 5:**
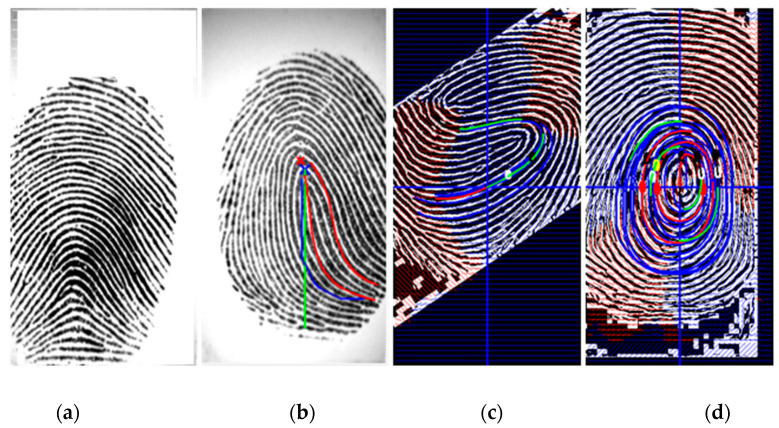
Fingerprint alignment: (**a**) number of cores = 0; (**b**) number of cores = 1; (**c**,**d**) number of cores = 2.

Next, we align the fingerprint image under the right-angle coordinate system based on the number and location of preliminary core points. Because fingerprints may have different numbers of cores, the first step in alignment is to adopt the preliminary Poincaré indexed positions as a reference. If the number of preliminary cores is 2, the image is rotated along the orientation calculated from the midpoint between the two cores. If the number of cores is equal to 1, the image is rotated along the direction calculated from the neighboring orientation of the core. If the number of cores is zero, the fingerprint is kept intact. The rotation angle is calculated as follows:

(14) $$\underset{j<y_{c}}{\emptyset }=\frac{1}{2}{tan}^{-1}\frac{\sum_{i\in \zeta }sin2O_{i,j}}{\sum_{i\in \zeta }cos2O_{i,j}},$$

where $O_{i,j}$ is the local orientation around a pixel and ζ is the core subregion of interest (COI) centered at the Poincaré index core point (*x_c_*, *y_c_*) with size of 60 × 60 pixels, which was determined to avoid possible variability near the boundary while one is fingerprinted by the reader. Fingerprint alignment is performed to make the pattern rotation-invariant and to reduce the false rejection rate. The rotations are given by the following Equation:

(15) $$\{\begin{array}{c} y^{\prime }=xsin\phi +ycos\phi \\ x^{\prime }=xcos\phi -ysin\phi \end{array},$$

and point (*x*, *y*) with orientation angle ϕ is mapped to point $\left(x^{\prime },y^{\prime }\right).$ Figure 5 shows some fingerprint alignment by our method with different numbers of cores.

**Figure 6:**
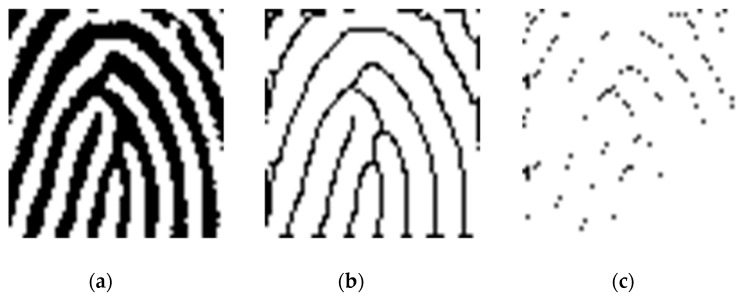
(**a**) COI subregion; (**b**)skeletonized ridges; (**c**) 2D wavelet extrema.

After alignment, the COI subregion with size of 60 × 60 pixels centered at the Poincaré’s detected point is further segmented from the aligned image. The COI then goes through a skeletonization process to peel off as many ridge pixels as possible without affecting the general shape of the ridge, as shown in Figure 6a, and is then transformed to a skeletonized ridge image, as shown in Figure 6b. The skeletonized ridge image is used to compute the wavelet extrema, as shown in Figure 6c.

Wavelet modulus maxima representations for two-dimensional signals were proposed by Mallat [33] as a tool for extracting information on singularities, which were considered to be among the most meaningful features for signal characterization. Most wavelet transform local extrema are actually modulus maxima (there are examples of signals for which the wavelet extrema and modulus representations are the same). The set of indices and the local maximum, denoted as M(f), and local minimum, denoted as m(f), of skeletonized ridge image *f* are defined as follows:

(16) M(f)={(z,f(z)):f(z−1)≤f(z)andf(z+1)≤f(z)},

(17) m(f)={(z,f(z)):f(z−1)≥f(z)andf(z+1)≥f(z)},

Where *z*∈*Z*. Similarly, the indices and values of wavelet transform extrema for an image *f* is defined as follows:

(18) $$E\left(f\right)=\left\{\left\{M\left(w_{j}\left(f\right)\right)\right\}\cup \left\{m\left(w_{j}\left(f\right)\right)\right\};j=1,2,\ldots ,J\right\},$$

where $w_{j}\left(f\right)$ is the 2D non-separable wavelet transform of image *f* at scale *j*, *j* = 1, 2,…, *J*. The SP of a fingerprint image can be found by extracting curvature primitives and discovering the location of these primitives in the subregion, as shown in Figure 6c.

**Figure 7:**
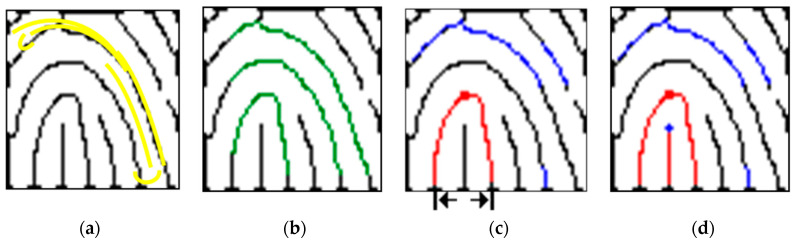
Core point detection based on wavelet extrema and Henry system. (**a**) Two 8-adjacency grids moving toward each other along the ridge curve indicated in yellow; (**b**) traced path of the ridge curve (green line: from left to right); (**c**) SP located at the lowest ridge curve (red square) and the area beneath (blue line: searching extrema from right to left); (**d**) SP detection in accordance with the Henry system (blue cross).

We find the exact location of the core point defined by the Henry system and trace the skeletonized ridge curves with 8-adjacency to explore wavelet extrema in 1-pixel increments by starting at 10 pixels apart from two sides. The highest extrema in the ridge curve correspond to core point candidates. We devise two 8-adjacency grids to locate the wavelet extrema (Figure 7a,b). Beginning from two opposite ends and moving toward the center of the subregion, the black-colored pixel of each grid is designated as the central point to trace. Based on this central point, the moving guideline is as follows: if the gray-level of the adjacent pixel is 0, then move toward that pixel, where the number shown in the grid indicates the moving sequence. This method enables one to follow the real track of the ridge curve. Whenever a singularity is detected, its location is noted. Figure 7c shows that it is common to find multiple core point candidates with small vertical displacements, and the area below the lowest ridge curve is circumscribed for locating the core point. In the Henry system, exact core point location can be performed as follows: (a) locate the topmost extrema in the innermost ridge curve if there is no rod; (b) otherwise, locate the top of the rods. The following equation summarizes this process:

(19) $$s=\{\begin{array}{c} \omega_{e,0},\;\;\;\;\;\;\;\;\;\;i=0\\ \omega_{e,\left(i/2\right)+\left(i\mathrm{mod}2\right)},\;\;i\geq 1 \end{array},$$

Where *s* is the determined core point, *i* is the number of rods below the innermost ridge curve, $\omega_{e,0}$ is the topmost extrema in the innermost ridge curve, and $\omega_{e,\left(i/2\right)+\left(i\mathrm{mod}2\right)}$ is the located rod extrema below the innermost ridge curve. Figure 7d presents an example marked with the blue cross.

## 4. Experimental Results and Discussion

In this section, to illustrate the effectiveness of our proposed method, we present some of the performed experiments using both FVC2002 DB1 and DB2 fingerprint databases. FVC2002 includes four databases, namely, DB1, DB2, DB3, and DB4, collected using different sensors or technologies that are widely used in practice. Each database is 110 fingers wide (w) and 8 impressions per finger deep (d) (880 fingerprints in all). Fingerprints from 101 to 110 (set B) have been made available to the participants to allow for parameter tuning before the submission of the algorithms. The benchmark is then constituted by fingers numbered from 1 to 100 (set A). Volunteers were randomly partitioned into three groups (30 persons each); each group was associated with a database and therefore to a different fingerprint scanner. Each volunteer was invited to present themselves at the collection place in three distinct sessions, with at least two weeks between each session. The forefinger and middle finger of both hands (in total, four fingers) of each volunteer were acquired by interleaving the acquisition of the different fingers to maximize differences in finger placement. No efforts were made to control image quality and the sensor platens were not systematically cleaned. In each session, four impressions were acquired of each of the four fingers of each volunteer. During the second session, individuals were requested to exaggerate the displacement (impressions 1 and 2) and rotation (impressions 3 and 4) of the finger without exceeding 35°. During the third session, fingers were alternatively dried (impressions 1 and 2) and moistened (impressions 3 and 4). The SPs of all fingerprints in the testing database were manually labeled beforehand to obtain the ground truth. For a ground-truth $SP\left(x_{0},y_{0}\right)$, if a detected SP(x,y) satisfies $\sqrt{{\left(x-x_{0}\right)}^{2}-{\left(y-y_{0}\right)}^{2}}<10$, it is said to be truly detected; otherwise, it is called a miss.

The singular point detection rate (SDR) is defined as the ratio of truly detected SPs to all ground-truth SPs:

(20) $$\mathrm{SDR}=\frac{Num\left(truly\;detected\;SPs\right)}{Num\left(groundtruth\;SPs\right)}\times 100\%.$$

The singular point miss rate (SMR) is defined as the ratio of the number of missed SPs to the number of all ground-truth SPs. The sum of the detection rate and miss rate is 100%:

(21) $$\mathrm{SMR}=\frac{Num\left(missed\;SPs\right)}{Num\left(groundtruth\;SPs\right)}\times 100\%=100-\mathrm{SDR}.$$

The singular point false alarm rate (SFR) is defined as the ratio of the number of falsely detected SPs to the total number of ground-truth *SP*s:

(22) $$\mathrm{SFR}=\frac{Num\left(falsely\;detected\;SPs\right)}{Num\left(groundtruth\;SPs\right)}\times 100\%.$$

The singular point correctly detected rate (SCR) is defined as the ratio of all truly detected SPs to all detected SPs in a fingerprint of all fingerprint images:

(23) $$\mathrm{SCR}=\frac{Num\left(truely\;detected\;SPs\right)}{Num\left(detected\;SPs\right)}\times 100\%.$$

**Figure 8:**
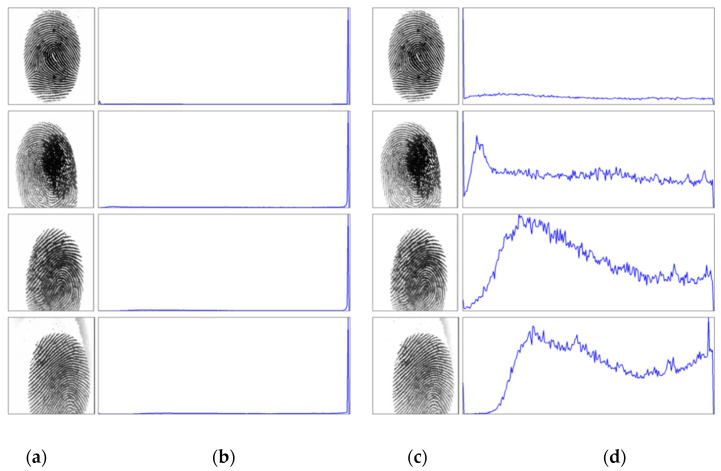
Results of our proposed method for the FVC2002 DB1 database. (**a**) Original fingerprint images; (**b**) histogram of Figure 8a; (**c**) equalized fingerprint images of Figure 8a; (**d**) histogram of Figure 8c.

**Figure 9:**
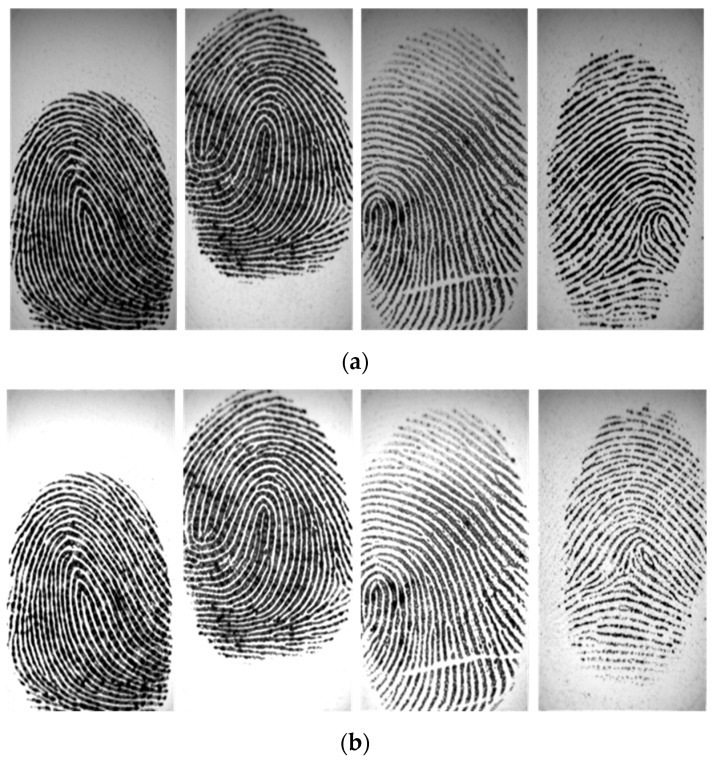
Results of our proposed method for the FVC2002 DB2 database. (**a**) Original fingerprint images and (**b**) equalized fingerprint images of Figure 9a.

First, the compensation weight coefficients are calculated by using Equation (4) and the equalized image,$f_{eq}$, having the same size as the original fingerprint image can be generated by Equation (5). Figure 8 and Figure 9 show example image results of the proposed method for FVC2002 DB1 and DB2, respectively. As shown in Figure 8c and Figure 9b, the background of the fingerprint image has been removed, thereby providing an image with nearly normal distribution. It also improves the clarity and continuity of ridge structures in the fingerprint image.

Then, we show the effectiveness by comparing the amount of information in our method and in the original fingerprint images by using the entropy of an image. The entropy of information *H* was introduced by Shannon [34] in 1948, and it can be calculated by the following equation:

(24) $$H=-\overset{255}{\underset{v=0}{\sum }}p_{i}\log_{2}p_{i},$$

where $p_{i}$ denotes the probability mass function of gray level *i*, and it is calculated as follows:

(25) $$p_{i}=\frac{Number\;of\;occurrences\;of\;intensity\;levels}{Number\;of\;intensity\;levels}.$$

In digital image processing, entropy is a measure of an image’s information content, which is interpreted as the average uncertainty of the information source. The entropy of an image can be used for measuring image visual aspects [35] or for gathering information to be used as parameters in some systems [36]. Entropy is widely used for measuring the amount of information within an image. Higher entropy implies that an image contains more information.

Entropy is measured to quantify the information produced from the enhanced image. For good enhancement, the entropy of the enhanced image should be close to that of the original image. This small difference between entropies of the original and the enhanced images indicates that the image details are preserved. It also shows that the histogram shape is maintained; thus, the saturation case can be avoided. Table 2 shows the entropy of equalized images compared with original images for each image shown in Figure 8 and Figure 9. The result shows that the equalized fingerprint images have smaller entropy while they are still close to the entropy of the original image. It means that our method can remove noise from the original image while retaining the structure of the fingerprint image.

**Figure 10:**
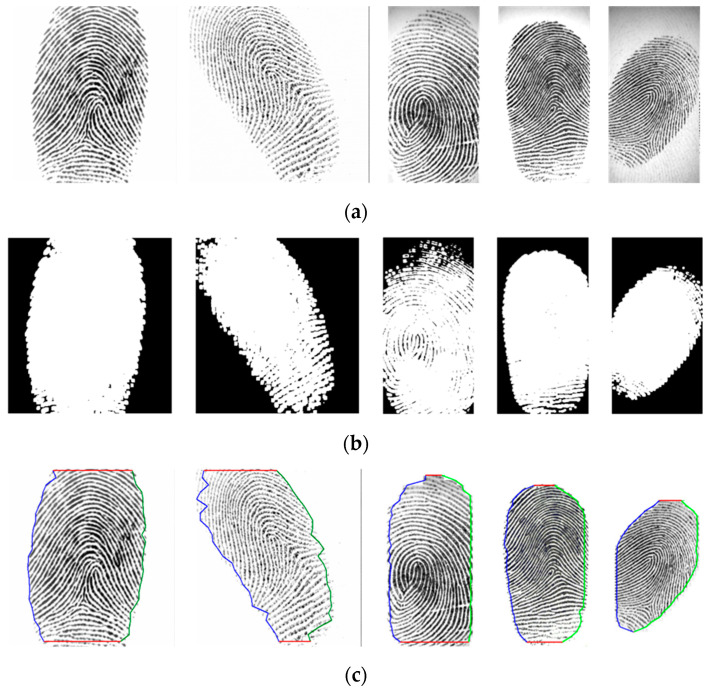
Binary images by using energy transformation for the FVC 2002 DB1 and DB2 databases. (**a**) Equalized images of five fingerprint images in the FVC 2002 database; (**b**) binary images of Figure 10a; (**c**) segmented images of Figure 10a.

**Figure 11:**
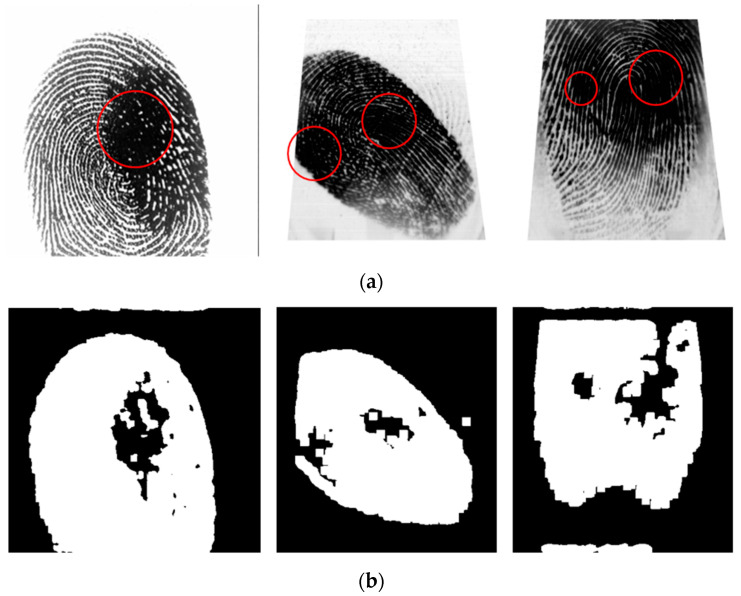
Blur detection result obtained by 2D non-separable wavelet entropy filtering for low-quality images: (**a**) original images and (**b**) blur detection results.

Next, the equalized fingerprint image was used to determine the contour and detected the blur region of the fingerprint, as discussed in Section 2.2 and Section 2.3. Figure 10 shows the binarized image obtained by applying Equation (8) to the equalized image. Based on the binary images, as shown in Figure 10b, we can detect the region of impression (ROI), and the contour of the fingerprint is acquired in a polygon, as shown in Figure 10c. Figure 11b presents the blur detection result obtained by 2D non-separable wavelet entropy filtering for low-quality images, as discussed in Section 2.3. In what follows, an ROI with a 30% blur region is considered to have bad quality, and its SP detection is not good enough.

**Figure 12:**
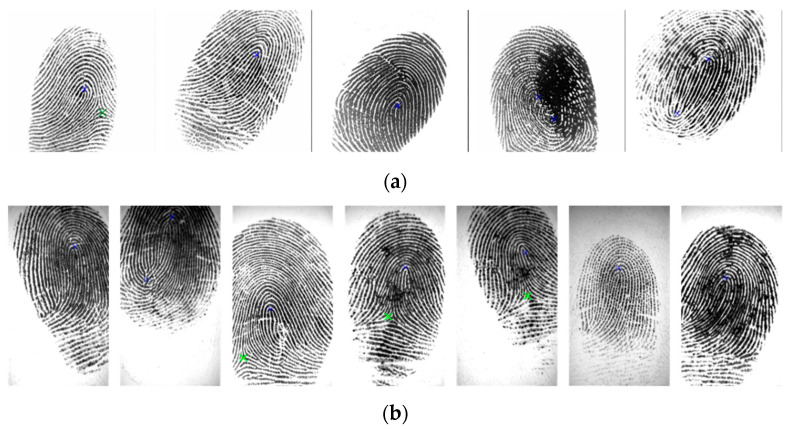
Truly detected SPs for the FVC2002 database (blue: core point; green: delta point) by our proposed method: (**a**) FVC2002 DB1 and (**b**) FVC2002 DB2 databases.

**Figure 13:**
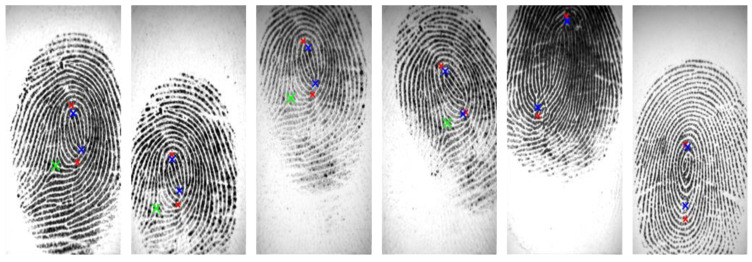
Some comparison results of SP detection for the FVC2002 database. The blue and green crosses indicate the core and delta points, respectively, detected by our proposed method, and the red cross indicates the core point detected by the Poincaré index method.

Our experiments were tested on the FVC2002 DB1_A and FVC2002 DB2_A databases. We compared the results of our proposed SP detection with results obtained using other methods, including a rule-based algorithm [5], Zhou’s algorithm [11], Tico’s algorithm [37], Ramo’s algorithm [38], and Chikkerur and Ratha’s algorithm [39]. In these methods, the singular points were measured on Euclidean distance. While no standard terms exist to define a correct detection, we devoted our attention in this research to a method for detecting a singular point precisely and followed the convention for adopting the 10-pixel deviation on the distance between the expected and the detected singular points to validate the performance of the proposed method. In addition, the singular point detection based on the Poincaré index method is sensitive for low-quality fingerprints. In this paper, we show that by combining a novel adaptive image enhancement, compact boundary segmentation, and NSDWT for localization, the detection of singular points is more robust. Moreover, a novel clustering algorithm by integrating wavelet frame entropy with region growing is introduced to evaluate the fingerprint image quality to validate the detected singular points. Table 3 and Table 4 show the correctly detected rate, detection rate, miss rate, and false alarm rate. The results in the tables indicate that our method not only has a higher correctly detected rate than other methods but also has a low false alarm rate. Figure 12 presents the results of truly detected SPs on the FVC2002 database; the core points and the delta points are closer as ground truth SPs. Figure 13 presents some comparison results of SP detection for the FVC2002 database using our proposed method and the Poincaré index method. In this figure, blue and green crosses indicate the core and delta points, respectively, detected by our proposed method, and the red cross indicates the core point detected by the Poincaré index method. The results show that the location of the SPs detected using our method is more accurate than those of the SPs detected using the Poincaré index method.

## 5. Conclusions

Because the conventional Poincaré index along the boundary of a given region equals the sum of the Poincaré indices of the core points within this region, it contains no information about the characteristics and cannot describe the core point completely. To solve this problem, we proposed an adaptive method to detect SPs in a fingerprint image. First, a novel fingerprint enhancement algorithm was proposed to considerably eliminate the background, thereby improving the clarity and continuity of ridge structures. Second, we demonstrated that the proposed algorithm could effectively detect low-quality regions with a high correct rate. Third, based on the threshold value, the proposed algorithm inspected and made a True/False decision about whether a detected SP was accepted. Experimental results demonstrate that the proposed algorithm effectively detects SPs and the results are better than those obtained by rule-based [5], Zhou [11], Tico [37], Ramo [38], and Chikkerur [39].

## Figures and Tables

**Table 1:** Mean and standard deviation of Gaussian distribution function in each database.

Database	Mean	Standard Deviation
FVC2002 DB1	0.84	0.24
FVC2002 DB2	0.50	0.18

**Table 2:** Entropy of equalized images compared with original images for each database.

	The Entropy of Image							
	FVC2002 DB1				FVC2002 DB2			
Original image	5.1222	5.5262	5.4171	5.3983	7.2446	7.1543	7.7012	7.5129
Equalized image	4.8028	5.3939	5.0496	4.9844	6.8401	7.0603	6.3322	6.2088

**Table 3:** Comparison results of various detection algorithms for the FVC2002 DB1-A fingerprint database.

Algorithm	SCR	SDR		SMR		SFR	
		Core	Delta	Core	Delta	Core	Delta
Tico’s [37]	58.50	90.27	55.49	9.83	44.51	10.78	80.20
Ramo’s [38]	53.54	92.19	68.42	7.81	31.58	8.47	46.15
Zhou’s [11]	88.88	95.78	96.98	4.22	3.02	2.27	9.97
Chikkerur’s [39]	85.13	95.89	92.75	4.11	7.25	6.93	8.16
Rule-based [5]	50.00	86.26	55.24	13.74	44.76	15.92	81.04
Proposed	90.72	92.43	97.25	7.57	2.75	1.41	3.07

**Table 4:** Comparison results of different detection algorithms for the FVC2002 DB2-A fingerprint database.

Algorithm	SCR	SDR		SMR		SFR	
		Core	Delta	Core	Delta	Core	Delta
Tico’s [37]	32.32	65.38	34.75	34.62	65.25	52.94	187.80
Ramo’s [38]	49.49	80.72	37.50	19.28	62.50	23.88	166.67
Zhou’s [11]	81.25	95.95	90.88	4.05	9.12	8.45	12.54
Chikkerur’s [39]	73.25	93.23	94.20	6.77	5.80	13.87	28.62
Rule-based [5]	56.57	73.86	37.61	26.14	62.39	35.40	165.85
Proposed	89.92	95.54	95.21	4.46	4.79	1.51	2.76
